# Estimated Vaccine Effectiveness for Pediatric Patients With Severe Influenza, 2015-2020

**DOI:** 10.1001/jamanetworkopen.2024.52512

**Published:** 2024-12-27

**Authors:** Kelsey M. Sumner, Leila C. Sahni, Julie A. Boom, Natasha B. Halasa, Laura S. Stewart, Janet A. Englund, Eileen J. Klein, Mary A. Staat, Elizabeth P. Schlaudecker, Rangaraj Selvarangan, Christopher J. Harrison, Geoffrey A. Weinberg, Peter G. Szilagyi, Monica N. Singer, Parvin H. Azimi, Benjamin R. Clopper, Heidi L. Moline, Emma K. Noble, John V. Williams, Marian G. Michaels, Samantha M. Olson

**Affiliations:** 1 Influenza Division, National Center for Immunization and Respiratory Diseases, US Centers for Disease Control and Prevention, Atlanta, Georgia; 2 Epidemic Intelligence Service, US Centers for Disease Control and Prevention, Atlanta, Georgia; 3 Department of Pediatrics, Baylor College of Medicine, Houston, Texas; 4 Texas Children’s Hospital, Houston; 5 Vanderbilt University Medical Center, Nashville, Tennessee; 6 Seattle Children’s Research Institute, Seattle, Washington; 7 Cincinnati Children’s Hospital Medical Center, University of Cincinnati College of Medicine, Cincinnati, Ohio; 8 University of Missouri–Kansas City School of Medicine, Children’s Mercy Kansas City, Kansas City; 9 University of Rochester School of Medicine and Dentistry, Rochester, New York; 10 UCLA Mattel Children's Hospital, Department of Pediatrics, University of California at Los Angeles; 11 University of California, San Francisco Benioff Children’s Hospital Oakland, Oakland; 12 Coronavirus and Other Respiratory Viruses Division, National Center for Immunization and Respiratory Diseases, US Centers for Disease Control and Prevention, Atlanta, Georgia; 13 Oak Ridge Institute for Science and Education, Oak Ridge, Tennessee; 14 UPMC Children’s Hospital of Pittsburgh, University of Pittsburgh School of Medicine, Pittsburgh, Pennsylvania; 15 Department of Pediatrics, University of Wisconsin School of Medicine and Public Health, Madison

## Abstract

**Importance:**

Increasing the understanding of vaccine effectiveness (VE) against levels of severe influenza in children could help increase uptake of influenza vaccination and strengthen vaccine policies globally.

**Objective:**

To investigate VE in children by severity of influenza illness.

**Design, Setting, and Participants:**

This case-control study with a test-negative design used data from 8 participating medical centers located in geographically different US states in the New Vaccine Surveillance Network from November 6, 2015, through April 8, 2020. Participants included children 6 months through 17 years of age who were hospitalized or presented to an emergency department (ED) with acute respiratory illness.

**Exposures:**

Receipt of at least 1 dose of the current season’s influenza vaccine.

**Main Outcomes and Measures:**

Demographic and clinical characteristics of patients presenting to the hospital or ED with or without influenza were recorded and grouped by influenza vaccination status. Estimated VE against severe influenza illness was calculated using multiple measures to capture illness severity. Data were analyzed between June 1, 2022, and September 30, 2023.

**Results:**

Among 15 728 children presenting for care with acute respiratory illness (8708 [55.4%] male; 13 450 [85.5%] 6 months to 8 years of age and 2278 [14.5%] 9-17 years of age), 2710 (17.2%) had positive influenza tests and 13 018 (82.8%) had negative influenza tests (controls). Of the influenza test–positive cases, 1676 children (61.8%) had an ED visit, 896 children (33.1%) required hospitalization for noncritical influenza, and 138 children (5.1%) required hospitalization for critical influenza. About half (7779 [49.5%]) of the children (both influenza test positive and test negative) were vaccinated. Receiving at least 1 influenza vaccine dose was estimated to have a VE of 55.7% (95% CI, 51.6%-59.6%) for preventing influenza-associated ED visits or hospitalizations among children of all ages. The estimated VE was similar across severity levels: 52.8% (95% CI, 46.6%-58.3%) for ED visits, 52.3% (95% CI, 44.8%-58.8%) for noncritical hospitalization, and 50.4% (95% CI, 29.7%-65.3%) for critical hospitalization.

**Conclusions and Relevance:**

Findings from this case-control study with a test-negative design involving children with a spectrum of influenza severity suggest that influenza vaccination protects children against all levels of severe influenza illness.

## Key Points

**Question:**

Among children in the US, how does influenza vaccine effectiveness (VE) vary by severity of influenza illness?

**Findings:**

In this case-control study with a test-negative design of 15 728 children with acute respiratory illness presenting for care in the US from 2015 to 2020, estimated VE for influenza vaccination was consistently at least 50% across all severity indicators. No difference in estimated VE was observed with increasing levels of influenza severity.

**Meaning:**

Findings from this case-control study suggest that children should receive influenza vaccination to protect against all levels of severe influenza illness.

## Teaser

This case-control study with a test-negative design estimates influenza vaccine effectiveness by severity of illness across 5 influenza seasons in children with acute respiratory illness presenting to medical centers located in different US states.

## Introduction

Influenza can lead to severe illness in children, with those less than 5 years of age or with an underlying medical condition at highest risk of severe outcomes.^1^ Annual influenza vaccination is recommended in the US for all persons 6 months or older and can reduce the risk of developing severe illness due to influenza virus infection^1^. However, influenza vaccine coverage in children is lower than the Healthy People 2030 Goal of 70%^2^ and has declined in recent years (from 64% in the 2019-2020 season to 48% in the 2023-2024 season as of February 2024).^3,4^ Uptake of other childhood vaccines, such as pertussis vaccines, increased after studies estimated high vaccine effectiveness (VE) against severe disease.^5^ Thus, improving an understanding of VE against severe influenza in children could increase uptake of influenza vaccination and improve vaccine policies globally.

Since the 2009 H1N1 pandemic, few studies have assessed VE against severe influenza,^6,7,8,9,10,11,12,13,14^ and those that have typically measured severity using a range of end points, including, symptom-based severity,^7^ hospitalization or emergency department (ED) visits,^12,13,14^ intensive care unit (ICU) admission,^8,9,10^ specific life-threatening outcomes,^6,9,10^ or death.^8,10^ While understanding VE against severe influenza in children is important, its determination using these end points is difficult because sample sizes have been small, with studies typically focused on 1 influenza season and a single health care facility.

Using influenza hospitalizations and ED visits captured in the US New Vaccine Surveillance Network (NVSN) from November 6, 2015, through April 8, 2020,^15^ we investigated VE against a spectrum of severe influenza illness in children aged 6 months through 17 years. Specifically, we described demographic and clinical characteristics by influenza vaccination status in inpatients or ED patients with influenza and estimated VE against severe influenza illness using multiple measures to capture illness severity across 5 influenza seasons.

## Methods

### Study Design

This case-control study estimated influenza VE against a spectrum of severe influenza illness in children using a test-negative design. This design entailed estimating the odds of vaccination in influenza test–positive patients experiencing acute respiratory illness (ARI) compared with influenza test–negative control patients with ARI.^16,17^Permission for study enrollment was provided electronically or in writing by each participating child’s parent or guardian, and assent was provided by the child when applicable. This study was approved by the US Centers for Disease Control and Prevention (CDC) and the participating institutional review boards. The Strengthening the Reporting of Observational Studies in Epidemiology (STROBE) reporting guideline was followed in the creation of this report.

### Patient Enrollment and Data Collection

To identify children with influenza and controls without influenza during the 2015 to 2016 through 2019 to 2020 influenza seasons, children 6 months to 17 years of age hospitalized or presenting to an ED with ARI at participating hospitals were enrolled in the NVSN. The NVSN is a population-based, active surveillance system that studies vaccine impact in children across participating hospitals located in Rochester, New York; Cincinnati, Ohio; Nashville, Tennessee; Kansas City, Missouri; Houston, Texas; Seattle, Washington; Oakland, California; and Pittsburgh, Pennsylvania.^18^ For Oakland, California, patients were enrolled for the 2015 to 2016 influenza season only, and in Pittsburgh, Pennsylvania, children were enrolled starting in the 2016 to 2017 season through 2019 to 2020 (eTable 1 in Supplement 1). During the 2015 to 2016 season, ED enrollments did not occur and were not included in this analysis; Kansas City, Missouri, did not have verified vaccination data for ED visits for the full study period and thus was not included in the ED analysis. In 2015 to 2016, hospitalized children were enrolled with a defined list of diagnoses.^13^ In later seasons, ARI was defined as the presence of at least 1 of the following symptoms within the last 14 days: fever, cough, otalgia, nasal congestion, rhinorrhea, pharyngitis, myalgia, vomiting, wheezing, dyspnea or rapid or shallow breathing, apnea, or brief resolved unexplained event. Symptoms and enrollment criteria mirrored previous NVSN VE studies.^12,13,14^

Children presenting to the ED or hospitalized with ARI were enrolled in the NVSN following informed consent if they were (1) a resident in the hospital catchment area, (2) ill for less than 14 days, and (3) able to be enrolled within 48 hours of admission for an ARI-related condition. Children were excluded from the final enrollment dataset if they (1) had fever and neutropenia associated with malignancy, (2) were readmitted to the hospital within 4 days of a prior discharge, (3) had been previously enrolled within 14 days before their current hospitalization, (4) were a recent transfer from a hospital where they had been admitted for over 48 hours, or (5) had a known nonrespiratory condition that caused their hospitalization.

At enrollment, an interview was conducted with each child’s parent or guardian to collect information regarding patient demographics, illness characteristics, and medical and vaccination history. Information about race and ethnicity (categorized as Black, non-Hispanic; Hispanic; White, non-Hispanic; and other)^19^ was collected by self-report during the enrollment interview or abstraction from the medical record as vaccine uptake may differ by race and ethnicity. The other race and ethnicity group included individuals who identified as American Indian or Alaska Native, Asian, Native Hawaiian or Other Pacific Islander, or multiple reported races or ethnicities; this group was consolidated due to small sample size. Standardized medical record review was performed for each child. Also at enrollment, research staff collected a midturbinate nasal swab or throat swab from each child. For some children, comparable residual respiratory specimens that had been collected for hospital or ED standard of care clinical testing were used instead of collecting a new research swab; these specimens were also subjected to research testing. Respiratory specimens were tested for influenza virus using the NxTAG Respiratory Pathogen Panel (Luminex; in California, Missouri, and Ohio),^20^ FilmArray Respiratory Panel (BioFire; in Washington),^21^ TaqMan Array microfluidic card (Applied Biosystems; in New York),^22,23^ or CDC real-time reverse transcription–polymerase chain reaction (RT-PCR) assays (in Pennsylvania, Tennessee, and Texas).^24,25^ The RT-PCR assays were performed to identify influenza A subtypes and B lineages. Additional details regarding participant enrollment and specimen collection have been previously published.^12,13,18^ Children aged 6 months through 17 years enrolled during the site-specific 2015 to 2016 through 2019 to 2020 influenza seasons were excluded from the analytic dataset to assess influenza VE if they (1) had inconclusive, discrepant, or missing influenza test results; (2) were enrolled 14 or more days after symptom onset or onset was unknown; (3) had unknown vaccination status; or (4) were missing relevant covariates (Figure).

**Figure.**
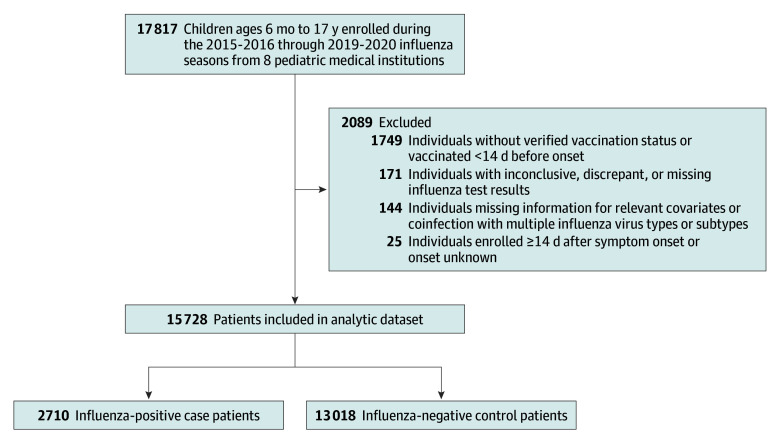
New Vaccine Surveillance Network Enrollment by Influenza Case and Control Status, 2015 to 2020

### Influenza Vaccination Status

Influenza vaccination status for each influenza season of the study was collected from all patients through the enrollment interview with their parent or guardian. Vaccination status was validated using state immunization information system records or by a health care professional.^12,13^ Children were classified as vaccinated if they received at least 1 dose of the current season influenza vaccine 14 or more days before symptom onset based on verified vaccination. A sensitivity analysis was conducted in which children were classified as fully vaccinated if they were 6 months to 8 years of age and received 2 influenza vaccine doses at least 14 days before symptom onset.^25^ If a child was 9 years of age or older, they were considered fully vaccinated if they received 1 influenza vaccine dose at least 14 days before symptom onset in the current season.

### Influenza Severity

Severity is a continuum of disease and condition; thus, VE was estimated against increasing levels of influenza severity. The severity levels ordered from least to most severe were (1) ED visit indicating an ED visit and not admitted to the hospital, (2) noncritical hospitalized illness indicating a hospitalization not requiring any of the critical hospitalized illness measures, and (3) critical hospitalized illness indicating an ICU admission, intubation, extracorporeal membrane oxygenation (ECMO) use, or death. The percentage of children vaccinated against influenza by each severity outcome was compared across influenza test–positive cases and test-negative controls. Length of hospital stay and ICU stay in influenza cases were also compared by vaccination status.

### Statistical Analysis

To estimate VE against the spectrum of severe influenza, we used multivariable logistic regression models comparing the odds of vaccination among influenza test–positive patients to influenza test–negative controls adjusted for age group, calendar time of illness onset, and study site. Calendar time of illness onset was defined as occurring before the peak, during the peak, and after the peak for each influenza season, with the influenza peak period of each season defined as being 2 weeks prior to through 2 weeks following the week with the most influenza cases. Confounders were identified a priori to be consistent with prior NVSN and other network multiseason VE studies.^12,26,27^ The adjusted odds ratio produced by the models was used to estimate VE with the following equation: (1 − the adjusted odds ratio) × 100.

In a sensitivity analysis, results were compared when stratified by presence of underlying conditions and when removing antiviral use before or during hospitalization to assess whether either impacted vaccine effectiveness against severe illness. A sensitivity analysis was conducted in which influenza VE was re-estimated after removing illnesses from influenza A(H3N2) virus, as the vaccine did not protect as well against A(H3N2) virus during some of the seasons included in this assessment.^28^ To compare differences in vaccine uptake across influenza cases, an additional analysis calculated the percentage of children vaccinated with specific underlying conditions across severity levels. All analyses were conducted in R, version 4.0.3 (R Project for Statistical Computing), using the package tidyverse.^29,30^ Data were analyzed from June 1, 2022, through September 30, 2023.

## Results

From November 6, 2015, to April 8, 2020, the NVSN enrolled 17 817 children with ARI during site-specific influenza seasons. A total of 15 728 children were included in the final analytic set, of whom 13 450 (85.5%) were 6 months to 8 years of age, and 2278 (14.5%) were 9 to 17 years of age; 8708 (55.4%) were male and 7020 (44.6%) were female; and 5167 (32.9%) were Black, non-Hispanic, 4190 (26.6%) were Hispanic, and 4909 (31.2%) were White, non-Hispanic (Figure and Table 1). Overall, 2710 children (17.2%) were influenza test positive and 13 018 children (82.8%) were influenza test negative. Of the influenza test–positive cases, 1676 children (61.8%) had an ED visit, 896 children (33.1%) had a noncritical inpatient visit, and 138 children (5.1%) had a critical inpatient visit (Table 2), including 138 children admitted to the ICU, 1 child with ECMO use, 35 children who were intubated, and 2 children who died. About half (7779 [49.5%]) of enrolled children in the analytic set (regardless of influenza case status) were vaccinated, and most of them (6791 [87.3%]) were 6 months through 8 years of age (Table 1). The percentage of test-positive influenza patients and test-negative controls who were vaccinated varied by season and measure of severe influenza illness (eTable 2 in Supplement 1). Unvaccinated patients with influenza had a longer length of hospital stay and more ICU days than those who had received influenza vaccine (eFigures 1 and 2 in Supplement 1). For patients who visited the ED with influenza, 461 (27.5%) had at least 1 underlying condition (Table 1). Among inpatient influenza cases, 621 children (60.1%) had at least 1 underlying condition and 519 children (50.2%) received influenza antiviral medications during admission (Table 1).

**Table 1.** Distribution of Demographics and Clinical Conditions for Participants by Influenza Status and Influenza Vaccination Status

Variable	Total participants, No. (%)	Influenza status, No. (%) of participants				Vaccination status, No. (%) of participants	
		Influenza negative		Influenza positive		Unvaccinated	Vaccinated (≥1 dose)
		Emergency Department	Inpatient	Emergency Department	Inpatient		
Total No.	15 728	4973	8045	1676	1034	7949	7779
Age							
6 mo to 8 y	13 450 (85.5)	4359 (87.7)	6923 (86.1)	1381 (82.4)	787 (76.1)	6659 (83.8)	6791 (87.3)
9-17 y	2278 (14.5)	614 (12.3)	1122 (13.9)	295 (17.6)	247 (23.9)	1290 (16.2)	988 (12.7)
Sex							
Female	7020 (44.6)	2318 (46.6)	3488 (43.4)	777 (46.4)	437 (42.3)	3589 (45.2)	3431 (44.1)
Male	8708 (55.4)	2655 (53.4)	4557 (56.6)	899 (53.6)	597 (57.7)	4360 (54.8)	4348 (55.9)
Race and ethnicity							
Black, non-Hispanic	5167 (32.9)	2094 (42.1)	1917 (23.8)	867 (51.7)	289 (27.9)	3284 (41.3)	1883 (24.2)
Hispanic	4190 (26.6)	1400 (28.2)	2152 (26.7)	448 (26.7)	190 (18.4)	1936 (24.4)	2254 (29.0)
White, non-Hispanic	4909 (31.2)	1118 (22.5)	3090 (38.4)	259 (15.5)	442 (42.7)	2066 (26.0)	2843 (36.5)
Other^a^	1410 (9.0)	335 (6.7)	866 (10.8)	98 (5.8)	111 (10.7)	643 (8.1)	767 (9.9)
Unknown	52 (0.3)	26 (0.5)	20 (0.2)	4 (0.2)	2 (0.2)	20 (0.3)	32 (0.4)
Underlying condition^b^							
No	8592 (54.6)	3563 (71.6)	3401 (42.3)	1215 (72.5)	413 (39.9)	4684 (58.9)	3908 (50.2)
Yes	7136 (45.4)	1410 (28.4)	4644 (57.7)	461 (27.5)	621 (60.1)	3265 (41.1)	3871 (49.8)
Insurance status							
Public	10 759 (68.4)	3846 (77.3)	4897 (60.9)	1353 (80.7)	663 (64.1)	5799 (73.0)	4960 (63.8)
Private	3851 (24.5)	796 (16.0)	2570 (31.9)	189 (11.3)	296 (28.6)	1473 (18.5)	2378 (30.6)
Both	329 (2.1)	42 (0.8)	242 (3.0)	17 (1.0)	28 (2.7)	126 (1.6)	203 (2.6)
None	675 (4.3)	251 (5.0)	290 (3.6)	97 (5.8)	37 (3.6)	482 (6.1)	193 (2.5)
Unknown	114 (0.7)	38 (0.8)	46 (0.6)	20 (1.2)	10 (1.0)	69 (0.9)	45 (0.6)
Influenza status							
Negative	13 018 (82.8)	NA	NA	NA	NA	6123 (77.0)	6895 (88.6)
Positive	2710 (17.2)	NA	NA	NA	NA	1826 (23.0)	884 (11.4)
Influenza type and subtype							
Negative	13 018 (82.8)	4973 (100)	8045 (100)	NA	NA	6123 (77.0)	6895 (88.6)
A(H3N2)	951 (6.0)	NA	NA	642 (38.3)	309 (29.9)	600 (7.5)	351 (4.5)
A(H1N1)pdm09	834 (5.3)	NA	NA	481 (28.7)	353 (34.1)	591 (7.4)	243 (3.1)
B	925 (5.9)	NA	NA	553 (33.0)	372 (36.0)	635 (8.0)	290 (3.7)
Duration of illness at enrollment, d							
0-2	8017 (51.0)	2929 (58.9)	3645 (45.3)	1047 (62.5)	396 (38.3)	4095 (51.1)	3922 (50.4)
3-4	4129 (26.3)	1096 (22.0)	2362 (29.4)	365 (21.8)	306 (29.6)	2016 (25.4)	2113 (27.2)
5-7	2725 (17.3)	710 (14.3)	1540 (19.1)	225 (13.4)	250 (24.2)	1404 (17.7)	1321 (17.0)
8-10	623 (4.0)	161 (3.2)	374 (4.6)	26 (1.6)	62 (6.0)	327 (4.1)	296 (3.8)
11-13	234 (1.5)	77 (1.5)	124 (1.5)	13 (0.8)	20 (1.9)	107 (1.3)	127 (1.6)
Received influenza antiviral medication before admission							
No	15 361 (97.7)	4927 (99.1)	7882 (98.0)	1634 (97.5)	918 (88.8)	7765 (97.7)	7596 (97.6)
Yes	313 (2.0)	40 (0.8)	123 (1.5)	41 (2.4)	109 (10.5)	154 (1.9)	159 (2.0)
Unknown	54 (0.3)	6 (0.1)	40 (0.5)	1 (0.1)	7 (0.7)	30 (0.4)	24 (0.3)
Received influenza antiviral medication during admission							
No	14 264 (90.7)	4861 (97.7)	7679 (95.5)	1209 (72.1)	515 (49.8)	7133 (89.7)	7131 (91.7)
Yes	1463 (9.3)	111 (2.2)	366 (4.5)	467 (27.9)	519 (50.2)	816 (10.3)	647 (8.3)
Unknown	1 (0.0)	1 (0.0)	0	0	0	0	1 (0.0)
Study site							
Cincinnati, Ohio	2183 (13.9)	774 (15.6)	956 (11.9)	346 (20.6)	107 (10.3)	1292 (16.3)	891 (11.5)
Houston, Texas	2840 (18.1)	543 (10.9)	2028 (25.2)	114 (6.8)	155 (15.0)	1246 (15.7)	1594 (20.5)
Kansas City, Missouri^c^	681 (4.3)	NA	592 (7.4)	NA	89 (8.6)	390 (4.9)	291 (3.7)
Nashville, Tennessee	3861 (24.5)	1960 (39.4)	1083 (13.5)	679 (40.5)	139 (13.4)	2250 (28.3)	1611 (20.7)
Oakland, California^d^	320 (2.0)	NA	297 (3.7)	NA	23 (2.2)	151 (1.9)	169 (2.2)
Pittsburgh, Pennsylvania^e^	2908 (18.5)	826 (16.6)	1543 (19.2)	226 (13.5)	313 (30.3)	1405 (17.7)	1503 (19.3)
Rochester, New York	1550 (9.9)	590 (11.9)	628 (7.8)	233 (13.9)	99 (9.6)	745 (9.4)	805 (10.3)
Seattle, Washington	1385 (8.8)	280 (5.6)	918 (11.4)	78 (4.7)	109 (10.5)	470 (5.9)	915 (11.8)
Influenza season of enrollment							
2015-2016^d^	1707 (10.9)	NA	1566 (19.5)	NA	141 (13.6)	741 (9.3)	966 (12.4)
2016-2017	3384 (21.5)	1309 (26.3)	1543 (19.2)	372 (22.2)	160 (15.5)	1628 (20.5)	1756 (22.6)
2017-2018	3661 (23.3)	1238 (24.9)	1744 (21.7)	465 (27.7)	214 (20.7)	2096 (26.4)	1565 (20.1)
2018-2019	3358 (21.4)	1305 (26.2)	1472 (18.3)	381 (22.7)	200 (19.3)	1509 (19.0)	1849 (23.8)
2019-2020	3618 (23.0)	1121 (22.5)	1720 (21.4)	458 (27.3)	319 (30.9)	1975 (24.8)	1643 (21.1)

Abbreviation: NA, not applicable.

^a^ The other race and ethnicity group included American Indian or Alaska Native, Asian, Native Hawaiian or Other Pacific Islander, and multiple reported races or ethnicities. This group was consolidated due to a small sample size.

^b^ Underlying conditions encompasses presence of at least 1 of the following conditions: respiratory, cardiovascular, neurological or neuromuscular, oncological or immunosuppressive, kidney or urological, gastrointestinal tract or hepatic, hematologic, genetic, endocrine or metabolic conditions or obesity.

^c^ Kansas City, Missouri, includes only hospitalized patients.

^d^ Only inpatient enrollments were included for the 2015 to 2016 season, which was the only season Oakland, California, was a site.

^e^ Pittsburgh, Pennsylvania was not a New Vaccine Surveillance Network site until the 2016 to 2017 season and onward.

**Table 2.** Estimated Influenza VE Across Measures of Influenza Illness Severity

Severe influenza illness measure	No. of vaccinated/total No. (%)		Estimated VE, % (95% CI)^a^
	Cases	Controls	
**All individuals**			
Overall	884/2710 (32.6)	6895/13 018 (53.0)	55.7 (51.6 to 59.6)
Influenza subtype			
Influenza A(H3N2)	351/951 (36.9)	6895/13 018 (53.0)	45.8 (37.6 to 52.9)
Influenza A(H1N1)pdm09	243/834 (29.1)	6895/13 018 (53.0)	64.9 (59.0 to 70.1)
Influenza B	290/925 (31.4)	6895/13 018 (53.0)	56.4 (49.6 to 62.4)
Age			
6 mo to 8 y	707/2168 (32.6)	6084/11 282 (53.9)	58.1 (53.7 to 62.1)
9-17 y	177/542 (32.7)	811/1736 (46.7)	42.6 (29.2 to 53.5)
**Emergency department visit**			
Overall	483/1676 (28.8)	2325/4973 (46.8)	52.8 (46.6 to 58.3)
Influenza subtype			
Influenza A(H3N2)	208/642 (32.4)	2325/4973 (46.8)	44.7 (33.8 to 54.0)
Influenza A(H1N1)pdm09	119/481 (24.7)	2325/4973 (46.8)	64.1 (55.4 to 71.3)
Influenza B	156/553 (28.2)	2325/4973 (46.8)	51.2 (40.7 to 60.1)
Age			
6 mo to 8 y	400/1381 (29.0)	2097/4359 (48.1)	55.4 (49.0 to 61.0)
9-17 y	83/295 (28.1)	228/614 (37.1)	35.4 (11.8 to 52.9)
**Noncritical influenza in inpatients**			
Overall	347/896 (38.7)	4570/8045 (56.8)	52.3 (44.8 to 58.8)
Influenza subtype			
Influenza A(H3N2)	128/271 (47.2)	4570/8045 (56.8)	30.7 (11.1 to 46.1)
Influenza A(H1N1)pdm09	101/301 (33.6)	4570/8045 (56.8)	64.4 (54.5 to 72.4)
Influenza B	118/324 (36.4)	4570/8045 (56.8)	55.0 (43.2 to 64.5)
Age			
6 mo to 8 y	269/696 (38.6)	3987/6923 (57.6)	55.5 (47.6 to 62.2)
9-17 y	78/200 (39.0)	583/1122 (52.0)	35.2 (10.9 to 53.1)
**Critical influenza in inpatients** ^b^			
Overall	54/138 (39.1)	4570/8045 (56.8)	50.4 (29.7 to 65.3)
Influenza subtype			
Influenza A(H3N2)	15/38 (39.5)	4570/8045 (56.8)	45.7 (−4.6 to 72.6)
Influenza A(H1N1)pdm09	23/52 (44.2)	4570/8045 (56.8)	39.4 (−5.6 to 65.7)
Influenza B	16/48 (33.3)	4570/8045 (56.8)	63.4 (33.4 to 80.6)
Age			
6 mo to 8 y	38/91 (41.8)	3987/6923 (57.6)	49.3 (22.5 to 67.1)
9-17 y	16/47 (34.0)	583/1122 (52.0)	51.5 (10.1 to 74.7)

Abbreviation: VE, vaccine effectiveness.

^a^ Logistic regression models were adjusted for age, illness onset in calendar time, and study site.

^b^ Critical inpatient visits were defined as hospitalization with intensive care unit admission, intubation, extracorporeal membrane oxygenation, or death.

Among influenza cases (ED or inpatient), 1082 children (39.9%) had at least 1 underlying medical condition. Respiratory conditions were most common among cases (670 of 2710 [24.7%]), followed by neurological or neuromuscular (202 of 2710 [7.5%]) and gastrointestinal or hepatic (164 of 2710 [6.1%]) conditions (Table 3). Influenza vaccination coverage by specific underlying condition varied by type of patient visit (ED, noncritical inpatient, or critical inpatient).

**Table 3.** Severity in Influenza Cases With Specific Underlying Conditions

Underlying condition	Total No. of influenza-positive participants with condition/total No. of influenza-positive participants (%)	No. of vaccinated influenza-positive participants with condition/total No. of influenza-positive participants with condition (%)								
		6-23 mo			2-8 y			9-17 y		
		ED visit	Noncritical inpatient visit	Critical inpatient visit	ED visit	Noncritical inpatient visit	Critical inpatient visit	ED visit	Noncritical inpatient visit	Critical inpatient visit
Any underlying condition	1082/2710 (39.9)	26/49 (53.1)	47/74 (63.5)	8/13 (61.5)	94/290 (32.4)	111/294 (37.8)	16/41 (39.0)	46/122 (37.7)	69/159 (43.4)	14/40 (35.0)
Cardiovascular^a^	127/2658 (4.8)	8/15 (53.3)	12/20 (60.0)	2/3 (66.7)	12/18 (66.7)	17/34 (50.0)	1/4 (25.0)	3/8 (37.5)	13/20 (65.0)	2/5 (40.0)
Endocrine^b^	57/2657 (2.1)	2/2 (100)	1/4 (25.0)	0/0	3/4 (75.0)	6/14 (42.9)	1/3 (33.3)	4/7 (57.1)	9/17 (52.9)	2/6 (33.3)
Gastrointestinal tract or hepatic^c^	164/2710 (6.1)	7/9 (77.8)	7/14 (50.0)	2/3 (66.7)	11/21 (52.4)	28/57 (49.1)	9/14 (64.3)	0/3 (<0.1)	22/31 (71.0)	6/12 (50.0)
Genetic or metabolic^d^	86/2662 (3.2)	3/3 (100)	7/12 (58.3)	1/1 (100)	5/7 (71.4)	15/31 (48.4)	4/8 (50.0)	2/3 (66.7)	11/13 (84.6)	3/8 (37.5)
Hematologic^e^	112/2660 (4.2)	5/5 (100)	8/11 (72.7)	2/3 (66.7)	12/26 (46.2)	16/33 (48.5)	1/2 (50.0)	1/7 (14.3)	8/23 (34.8)	1/2 (50.0)
Neurological or neuromuscular^f^	202/2710 (7.5)	4/5 (80.0)	15/19 (78.9)	4/4 (100)	9/20 (45.0)	24/66 (36.4)	11/20 (55.0)	4/10 (40.0)	25/40 (62.5)	8/18 (44.4)
Obesity^g^	150/713 (21.0)	NA	NA	NA	12/37 (32.4)	17/47 (36.2)	3/9 (33.3)	3/10 (30.0)	11/40 (27.5)	3/7 (42.9)
Oncological or immunosuppressive^h^	69/2656 (2.6)	3/5 (60.0)	0/0	0/1 (<0.1)	4/6 (66.7)	17/26 (65.4)	2/2 (100)	4/6 (66.7)	11/20 (55.0)	1/3 (33.3)
Kidney or urological^i^	34/2710 (1.3)	3/3 (100)	2/3 (66.7)	1/1 (100)	3/7 (42.9)	7/12 (58.3)	0/0	0/1 (<0.1)	6/7 (85.7)	0/0
Respiratory^j^	670/2710 (24.7)	11/24 (45.8)	19/30 (63.3)	7/10 (70.0)	69/196 (35.2)	66/159 (41.5)	12/27 (44.4)	41/95 (43.2)	50/105 (47.6)	9/24 (37.5)

Abbreviations: ED, emergency department; NA, not applicable.

^a^ A total of 52 observations were missing cardiovascular condition reporting.

^b^ A total of 53 observations were missing endocrine condition reporting.

^c^ No observations were missing gastrointestinal tract or hepatic condition reporting.

^d^ A total of 48 observations were missing genetic or metabolic condition reporting.

^e^ A total of 50 observations were missing hematologic condition reporting.

^f^ No observations were missing neurological or neuromuscular condition reporting.

^g^ Only captured for children 2 years of age or older with sufficient data to calculate body mass index. A child was defined as having obesity if their body mass index was in the 95th percentile or higher for the child’s age (months) and sex. Of 1970 observations, 1326 for children 2 years or older were missing obesity condition reporting.

^h^ A total of 54 observations were missing oncological or immunosuppressive condition reporting.

^i^ No observations were missing kidney or urological condition reporting.

^j^ No observations were missing respiratory condition reporting.

We estimated overall VE for the entire study period across measures of increasingly severe influenza illness. Overall estimated VE against influenza illness was 55.7% (95% CI, 51.6%-59.6%), with estimated VE differing for influenza A(H3N2) (45.8% [95% CI, 37.6%-52.9%]), A(H1N1)pdm09 (64.9% [95% CI, 59.0%-70.1%]), and B (56.4% [95% CI, 49.6%-62.4%]) viruses (Table 2). Estimated VE was higher for younger children (6 months to 8 years, 58.1% [95% CI, 53.7%-62.1%]) compared with older children (9-17 years, 42.6% [95% CI, 29.2%-53.5%]).

Estimated VE against ED visits was 52.8% (95% CI, 46.6%-58.3%), against hospitalization for noncritical illness was 52.3% (95% CI, 44.8%-58.8%), and against hospitalization for critical illness was 50.4% (95% CI, 29.7%-65.3%) (Table 2). The results had a similar pattern but generally higher point estimates when removing cases with influenza A(H3N2) virus and when comparing estimated VE in children considered fully vaccinated with children who received only 1 vaccine dose prior to illness onset among children 6 months to 8 years of age (eTable 3 in Supplement 1).

Analyses stratified by at least 1 underlying medical condition indicated that children with at least 1 underlying condition had lower VE point estimates across most measures of severity than children without underlying conditions, but the CIs overlapped (eTable 4 in Supplement 1). Specifically, among children with underlying conditions, influenza vaccination was estimated to be 36.4% (95% CI, 20.4%-49.3%) protective against ED visits, 46.3% (95% CI, 35.2%-55.5%) protective against noncritical inpatient visits, and 52.0% (95% CI, 26.8%-68.8%) protective against critical inpatient visits with influenza illness. When removing patients with antiviral medication use, point estimates slightly increased across measures of severity but had overlapping 95% CIs. Among children with no antiviral medication use before the medical encounter, influenza vaccination was estimated to be 54.6% (95% CI, 33.5%-69.3%) effective against critical inpatient visits and 53.3% (95% CI, 45.6%-60.0%) effective against noncritical inpatient visits. After removing patients with no antiviral medication use during the medical encounter, influenza vaccination was estimated to be 57.4% (95% CI, 20.5%-78.1%) effective against critical inpatient visits and 58.0% (95% CI, 48.8%-65.6%) effective against noncritical inpatient visits.

## Discussion

From 2015 to 2020, this case-control study found that receipt of at least 1 influenza vaccine dose was associated with just over a 50% reduction in influenza-associated pediatric ED visits or hospitalizations. The estimated influenza VE was highest among children 6 months to 8 years of age, ages at which children are at the highest risk for severe illness. We did not see a difference in estimated VE with increasing levels of influenza severity; however, influenza vaccination was consistently estimated to be 50% or more protective against medically attended illnesses for all influenza severity groups. These findings indicate that it is important for children to receive an annual influenza vaccine to protect against all levels of influenza illness severity, ranging from ED visits to critical hospitalized illness, during the influenza season. Only about 50% of enrolled children with ARI had received an influenza vaccine during the included enrollment seasons, falling short of the Healthy People 2030 Goal of 70%.^2^

Although some other studies^6,8^ have reported a difference in estimated influenza VE across measures of illness severity, when combining data from 5 influenza seasons, we did not observe similar findings. Prior work in children found higher point estimates for VE against pediatric ICU or high-acuity care admission with life-threatening compared with non–life-threatening illness during 1 season (2019-2020) predominated by influenza A(H1N1)pdm09 and B or Victoria viruses^6^; however, CIs for that study overlapped. Another study found higher estimated VE against critical vs noncritical hospitalizations during a season with mostly A(H1N1)pdm09 viruses, but it also had overlapping CIs as well as wide age ranges that included adults.^8^ Changes in predominant virus, differences in influenza seasons compared, and antigenic drift could be additional factors for our differing results from the literature.^31^ In the literature, VE against severe influenza has been assessed only during the 2019-2020 season, and we were unable to calculate season-specific VE across severity levels due to small sample sizes.^6^ The US experienced an influenza burden of low, moderate, and high severity with different virus predominance across the 5 seasons in this study.^32^ Additionally, season severity did not correlate with the reported pediatric VE against medically attended influenza,^33^ and the effect of antigenic drift on severity is unclear. Furthermore, the literature describing the effect of influenza type or subtype on influenza severity is limited.^34^ Thus, it is difficult to determine how these additional factors would influence VE against more severe manifestations of influenza. More research is needed to understand how influenza vaccines protect children against a gradient of severe outcomes and whether this varies by influenza type or subtype and season.

Preventive and treatment interventions (including vaccination and antiviral use) are emphasized in CDC recommendations for influenza, yet these tools could be used more (48% of children received influenza vaccine in the 2023-2024 season as of February 2024, and about 85% of hospitalized children received antiviral medication during the 2018-2019 season).^4,35^ Our data reflect these same issues. Antiviral use in this study was low in children, with only 50.2% of children with influenza receiving antiviral medication during hospital admission. The CDC recommends antiviral treatment as soon as possible for any patient with suspected or confirmed influenza who is hospitalized; has severe, complicated, or progressive illness; or is at higher risk for influenza complications. Previous work involving individuals hospitalized with influenza found lower antiviral receipt in hospitalized children 0 to 17 years of age than in hospitalized adults of any age.^35^ Influenza vaccine uptake was also low in children with underlying conditions presenting to the ED or admitted to the hospital in our study, despite some conditions increasing the risk of severe influenza.^36^ We were able to capture antiviral use only in an outpatient setting prior to the enrollment visit through self-reported data by the parent. Thus, there may have been some antiviral use prior to the enrollment that was not accounted for and could have led to the low antiviral use observed in the emergency department or inpatient setting. However, the suboptimal levels of antiviral use and influenza vaccine uptake in children with underlying conditions highlight the importance of early antiviral receipt and annual influenza vaccination to protect children at higher risk for severe influenza.

### Limitations

Our study had several limitations. First, the NVSN is an active surveillance network requiring patient contact and parent or guardian written informed consent for participation. Thus, we likely underenrolled children with severe influenza, especially children in critical condition, as the clinical care needs of severely ill (ie, unstable) patients would take precedence over logistical constraints of enrolling participants (eg, parent availability). As the network captured only 2 deaths among children with influenza over the 5-year study period, our severe-disease inpatient population may represent a milder spectrum of severe influenza outcomes than the true gradient of severe influenza disease that presents in these pediatric hospitals each season. This lack of catchment of the most severe patients may have contributed to similar VE estimates for inpatients with critical and with noncritical status in our study. However, this finding could also be within a reasonable range for estimated influenza deaths in this age group, according to seasonal CDC burden estimates and CDC surveillance of pediatric deaths. We also did not enroll and did not collect sufficient data on non-ED outpatients or individuals who did not seek care for influenza illnesses and were unable to comment on VE against milder or asymptomatic influenza virus infection. Additionally, VE against specific severity outcomes (eg, ECMO use only or death) could not be estimated due to a limited number of events. Yet a previous retrospective analysis of pediatric influenza-associated deaths from 2004 to 2012 found that most were among unvaccinated children and 43% had no high-risk condition.^37^ Moreover, we were unable to compare VE against severe illness across each influenza season due to limited numbers; however, we accounted for seasonal differences by adjusting the model for the influenza peak of each season. Due to a small sample size, we were unable to evaluate other potential confounders, and some CIs were wide. While study sites were geographically diverse, results may not be generalizable to the country as a whole.

## Conclusions

Overall, across a geographically diverse pediatric network including 5 influenza seasons of varying (low, moderate, and high) severity,^32^ this case-control study with a test-negative design found that receipt of at least 1 dose of influenza vaccine was estimated to be over 50% effective in protecting children against influenza-related ED and hospital visits. Vaccination is one of the most effective ways to protect children against influenza and its complications, including severe illness and hospitalization. Improving vaccine uptake in children may reduce influenza illness and, subsequently, ED and hospital visits in a time of increased respiratory virus co-circulation.

## Notes

Supplement 1. **eTable 1.** Site-Specific Influenza Seasons **eTable 2.** Distribution of Influenza Severity Outcomes Across Seasons **eTable 3.** Influenza Vaccine Effectiveness Across Measures of severe Influenza Illness—Removing A(H3N2) Illnesses **eTable 4.** Influenza Vaccine Effectiveness Across Measures of Severe Influenza Illness Stratified by Underlying Conditions and Antiviral Use **eFigure 1.** Length of Hospital Stay Among Children With Influenza Stratified by Vaccination Status **eFigure 2.** Length of Intensive Care Unit (ICU) Stay Among Children With Influenza Stratified by Vaccination Status

Supplement 2. **Nonauthor Collaborators.** The New Vaccine Surveillance Network Collaborators

Supplement 3.
Data Sharing Statement
