# Supplementary material for: Estimated Vaccine Effectiveness for Pediatric Patients With Severe Influenza, 2015-2020
Source: JAMA Netw Open. 2024 Dec 27;7(12):e2452512. doi: 10.1001/jamanetworkopen.2024.52512 (PMC11681373; doi:10.1001/jamanetworkopen.2024.52512)
Supplement: Supplement 1. — eTable 1. Site-Specific Influenza Seasons eTable 2. Distribution of Influenza Severity Outcomes Across Seasons eTable 3. Influenza Vaccine Effectiveness Across Measures of severe Influenza Illness—Removing A(H3N2) Illnesses eTable 4. Influenza Vaccine Effectiveness Across Measures of Severe Influenza Illness Stratified by Underlying Conditions and Antiviral Use eFigure 1. Length of Hospital Stay Among Children With Influenza Stratified by Vaccination Status eFigure 2. Length of Intensive Care Unit (ICU) Stay Among Children With Influenza Stratified by Vaccination Status [file jamanetwopen-e2452512-s001.pdf]

Supplementary Online Content

Sumner KM, Sahni LC, Boom JA, et al. Vaccine effectiveness for pediatric patients with severe influenza, 2015-2020. *JAMA Netw Open*. 2024;7(12):e2452512. doi:10.1001/jamanetworkopen.2024.52512

eTable 1. Site-specific influenza seasons

eTable 2. Distribution of influenza severity outcomes across seasons

eTable 3. Influenza vaccine effectiveness across measures of severe influenza illness – removing A(H3N2) illnesses

eTable 4. Influenza vaccine effectiveness across measures of severe influenza illness stratified by underlying conditions and antiviral use

eFigure 1. Length of hospital stay among children with influenza stratified by vaccination status

eFigure 2. Length of intensive care unit (ICU) stay among children with influenza stratified by vaccination status

This supplementary material has been provided by the authors to give readers additional information about their work.

© 2024 Sumner KM et al. *JAMA Network Open*.

eTable 1. Site-specific influenza seasons

Season	Site							
	Cincinnati, OH	Houston, TX	Kansas City, MO	Nashville, TN	Oakland, CA	Pittsburgh, PA	Rochester, NY	Seattle, WA
2015–2016	1/21/2016–4/29/2016	12/28/2015–6/16/2016	1/18/2016–4/18/2016	3/3/2016–6/17/2016	12/7/2015–5/19/2016	Not a site	3/7/2016–5/9/2016	11/6/2015–5/29/2016
2016–2017	1/7/2017–5/6/2017	12/28/2016–5/3/2017	12/29/2016–5/9/2017	12/16/2016–5/31/2017	Not a site	12/14/2016–4/19/2017	12/22/2016–5/15/2017	12/8/2016–6/9/2017
2017–2018	11/22/2017–6/12/2018	11/1/2017–4/21/2018	12/18/2017–4/3/2018	10/19/2017–4/26/2018	Not a site	11/28/2017–5/3/2018	11/27/2017–5/3/2018	12/7/2017–5/3/2018
2018–2019	1/15/2019–5/7/2019	11/7/2018–6/20/2019	11/13/2018–5/7/2019	11/7/2018–6/21/2019	Not a site	12/21/2018–5/31/2019	12/10/2018–4/24/2019	11/13/2018–5/13/2019
2019–2020	11/22/2019–3/26/2020	10/14/2019–3/19/2020	11/26/2019–4/8/2020	9/29/2019–3/19/2020	Not a site	11/8/2019–3/20/2020	11/11/2019–3/20/2020	11/4/2019–3/24/2020

© 2024 Sumner KM et al. *JAMA Network Open*.

eTable 2. Distribution of influenza severity outcomes across seasons

Influenza severity measure	No. vaccinated/Total no. (%)	
	Influenza test positive participants	Influenza test negative participants
**2015–2016: Low severity season for children[a]**		
All individuals	57/141 (40.4)	909/1,566 (58.0)
Emergency department visit[b]	NA	NA
Non-critical inpatient visit	55/132 (41.7)	909/1,566 (58.0)
Critical inpatient visit	2/9 (22.2)	909/1,566 (58.0)
**2016–2017: Moderate severity season for children[a]**		
All individuals	175/532 (32.9)	1,581/2,852 (55.4)
Emergency department visit	116/372 (31.2)	687/1,309 (52.5)
Non-critical inpatient visit	54/147 (36.7)	894/1,543 (57.9)
Critical inpatient visit	5/13 (38.5)	894/1,543 (57.9)
**2017–2018: High severity season for children[a]**		
All individuals	202/679 (29.7)	1,363/2,982 (45.7)
Emergency department visit	120/465 (25.8)	483/1,238 (39.0)
Non-critical inpatient visit	71/180 (39.4)	880/1,744 (50.5)
Critical inpatient visit	11/34 (32.4)	880/1,744 (50.5)
**2018–2019: Moderate severity season children[a]**		
All individuals	222/581 (38.2)	1,627/2,777 (58.6)
Emergency department visit	128/381 (33.6)	688/1,305 (52.7)
Non-critical inpatient visit	76/167 (45.5)	939/1,472 (63.8)
Critical inpatient visit	18/33 (54.5)	939/1,472 (63.8)
**2019–2020: High severity season children[a]**		
All individuals	228/777 (29.3)	1,415/2,841 (49.8)
Emergency department visit	119/458 (26.0)	467/1,121 (41.7)
Non-critical inpatient visit	91/270 (33.7)	948/1,720 (55.1)
Critical inpatient visit	18/49 (36.7)	948/1,720 (55.1)

Abbreviations: NA, Not applicable

[a] Pediatric seasonal influenza severity defined by CDC (Centers for Disease Control and Prevention. How CDC Classifies Flu Severity. https://www.cdc.gov/flu/php/surveillance/past-seasons.html~~https://www.cdc.gov/flu/about/classifies-flu-severity.htm~~)

[b] NVSN emergency department enrollments were not conducted in the 2015-2016 season.

© 2024 Sumner KM et al. *JAMA Network Open*.

eTable 3. Influenza vaccine effectiveness across measures of severe influenza illness – removing A(H3N2) illnesses

Severe influenza illness measure	No. vaccinated (≥1 dose)/Total no. (%)		VE[a] (≥1 dose) % (95% CI)	VE[a] (Fully vaccinated)[b] % (95% CI)
	Cases	Controls		
**All individuals**				
**Overall**	533/1759 (30.3)	6895/13018 (53.0)	60.7 (56.1, 64.8)	60.9 (55.8, 65.5)
**Age**				
6 months–8 years	432/1437 (30.1)	6084/11282 (53.9)	62.9 (58.2, 67.2)	63.9 (58.5, 68.7)
9–17 years	101/322 (31.4)	811/1736 (46.7)	47.6 (32.2, 59.8)	
**Emergency department visit**				
**Overall**	275/1034 (26.6)	2325/4973 (46.8)	57.7 (50.8, 63.7)	56.1 (47.8, 63.3)
**Age**				
6 months–8 years	228/866 (26.3)	2097/4359 (48.1)	60.7 (53.7, 66.8)	60.1 (51.4, 67.4)
9–17 years	47/168 (28.0)	228/614 (37.1)	36.4 (7.3, 56.9)	
**Non-critical inpatient visit**				
**Overall**	219/625 (35.0)	4570/8045 (56.8)	59.7 (52.1, 66.2)	60.0 (51.6, 67.0)
**Age**				
6 months–8 years	174/501 (34.7)	3987/6923 (57.6)	62.3 (54.3, 69.0)	63.3 (54.4, 70.7)
9–17 years	45/124 (36.3)	583/1122 (52.0)	45.0 (18.6, 63.2)	
**Critical inpatient visit[c]**				
**Overall**	39/100 (39.0)	4570/8045 (56.8)	52.0 (27.9, 68.5)	53.5 (27.7, 70.6)
**Age**				
6 months–8 years	30/70 (42.9)	3987/6923 (57.6)	47.6 (15.2, 68.0)	48.7 (12.5, 70.7)
9–17 years	9/30 (30.0)	583/1122 (52.0)	60.4 (13.8, 83.2)	

Abbreviations: CI, confidence interval; VE, vaccine effectiveness

[a] Logistic regression models were adjusted for age, illness onset in calendar time, and study site.

[b] A child was considered fully vaccinated if they were 6 months–8 years of age and received 2 influenza vaccine doses ≥14 days before symptom onset. If a child was 9 years of age or older, they were considered fully vaccinated if they received 1 influenza vaccine dose ≥14 days before symptom onset in the current season (Grohskopf LA, et al. "Prevention and Control of Seasonal Influenza with Vaccines: Recommendations of the Advisory Committee on Immunization Practices - United States, 2023-24 Influenza Season.") Individuals who were partially vaccinated were excluded.

[c] Critical inpatient visits were defined as hospitalization with intensive care unit admission, intubation, ECMO, or death.

© 2024 Sumner KM et al. *JAMA Network Open*.

eTable 4. Influenza vaccine effectiveness[a] across measures of severe influenza illness stratified by underlying conditions and antiviral use

	Original	Underlying conditions[b]		Antiviral use reported before the enrollment[c]		Antiviral use during the medical encounter[d]	
		No	Yes	No	Yes	No	Yes
**All individuals**	55.7 (51.6, 59.6)	60.2 (55.0, 64.8)	47.2 (39.6, 54.0)	56.7 (52.5, 60.6)	----	61.8 (57.2, 65.9)	41.7 (26.2, 54.1)
**ED visit**	52.8 (46.6, 58.3)	58.2 (51.4, 64.0)	36.4 (20.4, 49.3)	53.1 (46.9, 58.7)	----	57.7 (51.1, 63.4)	33.3 (-3.8, 57.0)
**Non-critical inpatient visit**	52.3 (44.8, 58.8)	60.0 (49.5, 68.5)	46.3 (35.2, 55.5)	53.3 (45.6, 60.0)	----	58.0 (48.8, 65.6)	38.4 (16.2, 54.8)
**Critical inpatient visit[e]**	50.4 (29.7, 65.3)	47.8 (2.3, 73.0)	52.0 (26.8, 68.8)	54.6 (33.5, 69.3)	----	57.4 (20.5, 78.1)	45.7 (12.4, 66.6)

Abbreviations: CI, confidence interval; VE, vaccine effectiveness; ED, emergency department

[a] Logistic regression models were adjusted for age, illness onset in calendar time, and study site.

[b] Underlying conditions encompasses presence of at least one of the following conditions: respiratory, cardiovascular, neurological/neuromuscular, oncologic/immunosuppressive, hematologic, kidney/urologic, gastrointestinal/hepatic, endocrine, genetic/metabolic, or obesity.

[c] Based on data self-reported by the parent/guardian answering the question whether the child has taken any influenza-specific antiviral medications for this illness before enrollment. The sample size was too small to calculate VE among those reporting antiviral use before the enrollment.

[d] Based on data from the chart review of whether the child has taken any influenza-specific antiviral medications for this illness during the medical encounter.

[e] Severe hospitalized influenza was defined as intensive care unit admission, intubation, extracorporeal membrane oxygenation~~ECMO~~, or death.

© 2024 Sumner KM et al. *JAMA Network Open*.

eFigure 1. Length of hospital stay among children with influenza stratified by vaccination status. The length of hospital stay among children with influenza that had an inpatient visit is shown for whether the child was vaccinated (dark blue) or unvaccinated (light blue) with the current season's influenza vaccine.

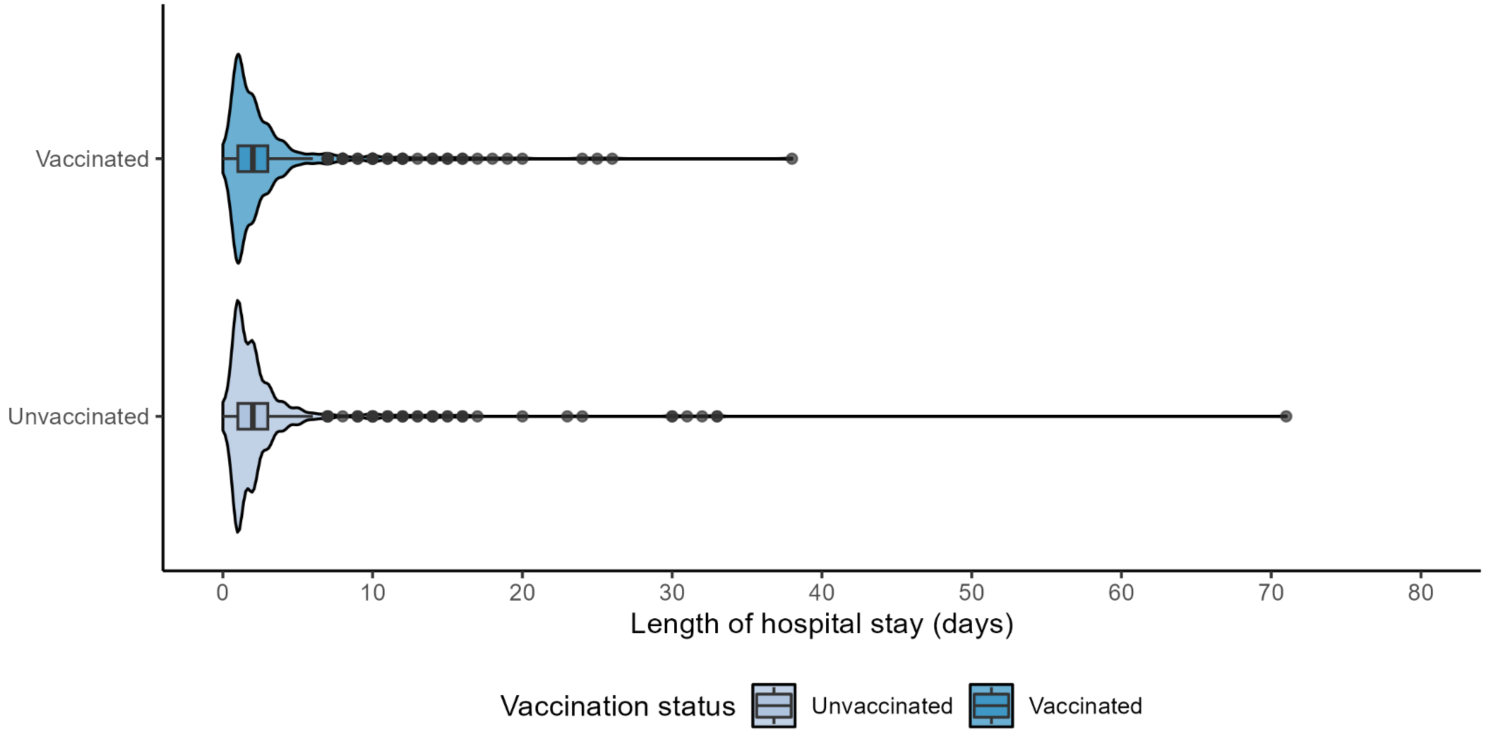

© 2024 Sumner KM et al. *JAMA Network Open*.

eFigure 2. Length of intensive care unit (ICU) stay among children with influenza stratified by vaccination status. The length of ICU stay among children with influenza that were admitted to the ICU is shown for whether the patient was vaccinated (dark blue) or unvaccinated (light blue) with the current season's influenza vaccine. Two persons did not have their length of ICU stay recorded and were omitted from this plot.

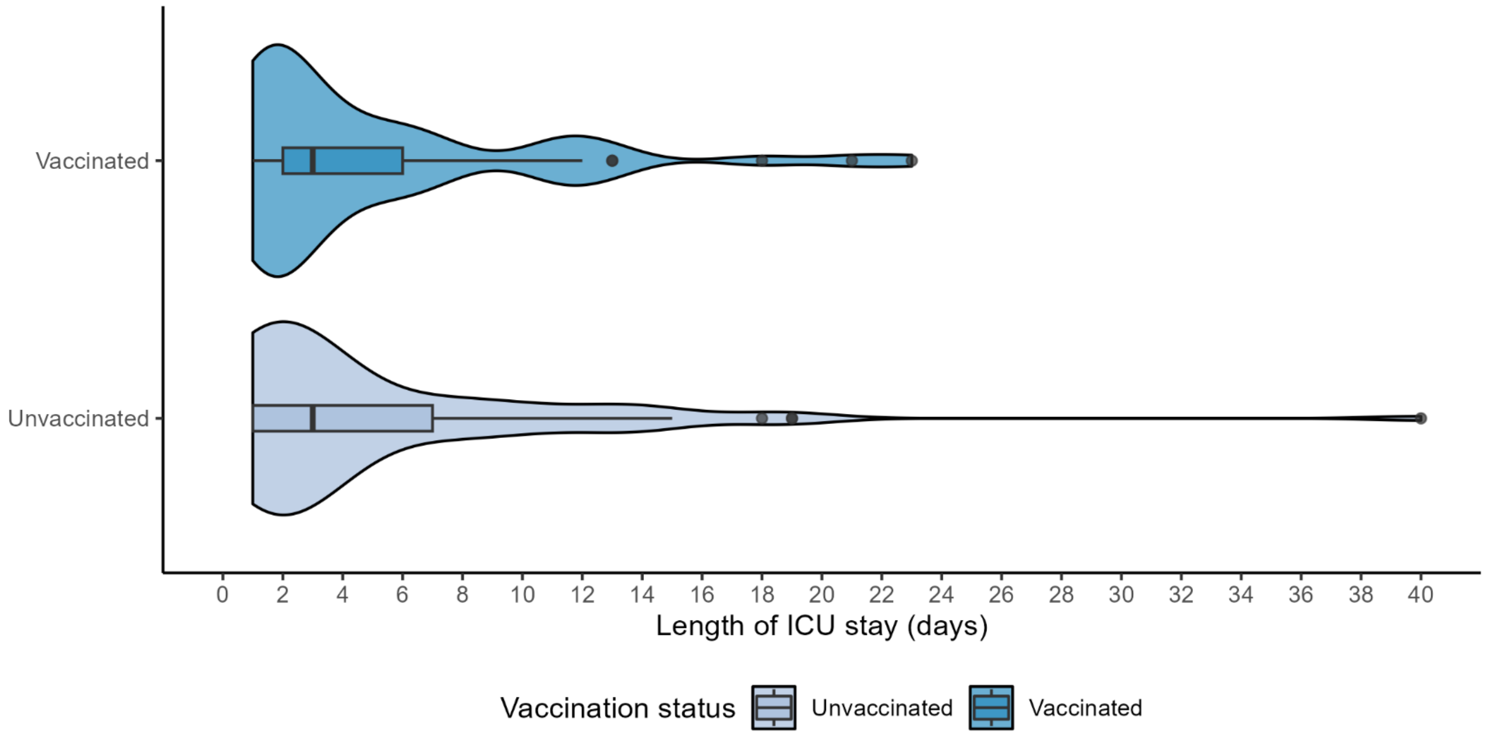

© 2024 Sumner KM et al. *JAMA Network Open*.
