# Supplementary material for: Estimated Vaccine Effectiveness for Pediatric Patients With Severe Influenza, 2015-2020
Source: JAMA Netw Open. 2024 Dec 27;7(12):e2452512. doi: 10.1001/jamanetworkopen.2024.52512 (PMC11681373; doi:10.1001/jamanetworkopen.2024.52512)
Supplement: Supplement 2. — Nonauthor Collaborators. The New Vaccine Surveillance Network Collaborators [file jamanetwopen-e2452512-s002.pdf]

Supplemental Online Content: Nonauthor Collaborators
*First name, last name, and suffix (if applicable) are required and will appear in PubMed.

***Group Name(s): New Vaccine Surveillance Network Collaborators**							
***First Name and Middle Initial(s)**	***Last Name**	***Suffix (eg, Jr, III)**	Academic Degrees	Institution	Location (city, state/province, country)	Role or Contribution, eg, chair, principal investigator	Group (if more than 1 Group listed in the byline) and/or Subgroup (eg, Steering Committee)
Jennifer E	Schuster		MD	University of Missouri-Kansas City School of Medicine & Children's Mercy Hospital	Kansas City, Missouri	PI	New Vaccine Surveillance Network Collaborators
