# Supplementary material for: Estimated Vaccine Effectiveness for Pediatric Patients With Severe Influenza, 2015-2020
Source: JAMA Netw Open. 2024 Dec 27;7(12):e2452512. doi: 10.1001/jamanetworkopen.2024.52512 (PMC11681373; doi:10.1001/jamanetworkopen.2024.52512)
Supplement: Supplement 3. — Data Sharing Statement [file jamanetwopen-e2452512-s003.pdf]

Data Sharing Statement

Sumner. Vaccine Effectiveness for Pediatric Patients With Severe Influenza, 2015-2020. *JAMA Netw Open*. Published December 27, 2024. doi:10.1001/jamanetworkopen.2024.52512

Data
Data available: No

Additional Information
Explanation for why data not available: The data that support the findings of this study are available from the corresponding author upon reasonable request. The data are not publicly available due to privacy or ethical restrictions.
